# Microbial next generation DNA sequencing of aspirated synovial fluid shows concordance with ICM criteria biomarkers for diagnosing periprosthetic joint infection in hip and knee arthroplasty

**DOI:** 10.3389/fmicb.2026.1816780

**Published:** 2026-05-11

**Authors:** Craig D. Tipton, Jacob Ancira, Saad Tarabichi, Alisina Shahi, Kayla Jarvis, Khalid Omeir, Nicholas Sanford, Nick A. Tallman, Jennifer White, Caleb D. Phillips, Javad Parvizi, Edward J. McPherson

**Affiliations:** 1 MicroGen DX, Lubbock, TX, United States; 2 Department of Biological Sciences, Texas Tech University, Lubbock, TX, United States; 3 Department of Orthopedic Surgery, Mayo Clinic Arizona, Phoenix, AZ, United States; 4 Department of Orthopedic Surgery, University of Texas Health Science Center at Houston, Houston, TX, United States; 5 International Joint Center, Acibadem University Hospital, Istanbul, Türkiye; 6 Department of Orthopedic Surgery, David Geffen School of Medicine, University of California Los Angeles, Los Angeles, CA, United States

**Keywords:** 16S rRNA sequencing, diagnostic criteria, ICM-18, ITS sequencing, next generation sequencing, PJI, synovial biomarkers, synovial fluid

## Abstract

**Background:**

The diagnosis of periprosthetic joint infection (PJI) is facilitated by consensus identification of synovial biomarkers, which may be aided by targeted microbial next generation sequencing (NGS) of synovial fluid. The primary objective of the study was to evaluate NGS performance across 3 years to ICM 2018 minor criteria for PJI.

**Methods:**

Synovial fluid specimens submitted from 2020–2022 by outpatient surgical clinics to MicroGenDX for matched synovial biomarker and NGS analysis were selected for retrospective analysis. Synovial biomarkers tested included C-reactive protein (CRP), white blood cell (WBC) count, and polymorphonuclear (PMN) leukocyte percentage. Synovial fluid analysis compared NGS microbial positivity with positive incidence of PJI determined by scoring of synovial biomarkers using ICM 2018 minor criteria for infection.

**Results:**

The overall sensitivity, specificity, and accuracy of NGS to ICM diagnosed PJI across 2,011 specimens was 76.4% [95% CI, 0.723–0.801], 92.3% [0.91–0.94], and 88.7% [0.87–0.90], respectively. When comparing the diagnostic performance of NGS and individual biomarkers to infection, NGS was more specific to PJI than synovial CRP (specificity = 0.894, 95% CI: 0.88–0.91), but not PMN or WBC. NGS was positive in 7.7% of ICM negative samples. NGS positive: ICM negative samples were associated with significantly elevated synovial PMN (*p* = 0.001) and WBC (*p* < 0.0001) compared to NGS negative: ICM negative samples. Across all samples, NGS positivity was associated with significantly elevated results for all tested biomarkers (*p* < 0.0001). Eight bacterial species dominated the composition in 68% of samples, whereas 46 different microbes were dominant in the remaining third.

**Conclusion:**

Microbial targeted NGS positivity was concordant with ICM minor criteria for PJI and should be considered a useful tool for diagnosis. There is a low risk of false positive detections comparable to ICM biomarkers. Elevated biomarkers in NGS positive: ICM negative samples may indicate infection occurring that is poorly captured by the three measured synovial biomarkers. Uncommon species are collectively common to PJI and NGS is uniquely positioned to detect such species as they are frequently missed by conventional microbiological testing, including culture and quantitative PCR. These results suggest formal diagnostic schemes would benefit from the addition of NGS as a diagnostic criterion.

## 1 Introduction

Periprosthetic joint infection (PJI) is a devastating complication of joint arthroplasty (JA), requiring aggressive salvage surgeries (Bozic et al., 2009), leading to increased mortality rates (Xu et al., 2023) and worsening quality of life (Xu et al., 2023). No single test is sufficiently diagnostic for all PJI and expert panels continuously seek to improve consensus guidelines defining PJI based on combinations of clinical presentation, inflammatory biomarkers, and microbiological testing. The International Consensus Meeting 2018 (ICM-18) definition (Parvizi and Gehrke, 2014; Parvizi et al., 2018) is one widely accepted scheme used for diagnosis, which is composed of major and minor criteria defining PJI (Parvizi et al., 2018). Major criteria include two phenotypically matching positive cultures or an open sinus tract communicating with the joint. If either criterion is observed, the joint is deemed infected, but major criteria are not met in up to 29% of cases (Sigmund et al., 2022).

Minor diagnostic criteria were developed to aid PJI confirmation based on a cumulative scoring index, including a single positive culture, established thresholds for serum or synovial fluid biomarkers, and histological tissue analysis at the time of debridement. The minor criteria are weighted to arrive at a diagnosis for PJI. Klement et al. reported that minor and major criteria agreed in 88% of cases for the diagnosis of PJI (Klement et al., 2018). PJI diagnosed only by minor criteria have similar success rates when treated and are thus no less significant while lacking major criteria (Klement et al., 2018). The integration of synovial fluid biomarkers is advantageous as synovial fluid is relatively easy to obtain during clinical visits, allowing earlier diagnosis of PJI, and can potentially identify infecting microbes from a single sample (Solarino et al., 2022; Cipriano et al., 2012). However, culture-negative PJI is a common problem occurring in the range of 28–46% (Klement et al., 2018; Goswami et al., 2022) of PJI cases and particularly challenging because the physician is given no guidance on antimicrobial therapy (Goswami et al., 2022; Mortazavi et al., 2011). Thus, techniques improving preoperative detection of microbes are advantageous, and can improve the fidelity of diagnostic criteria.

Molecular techniques for microbial identification continue to increase in popularity because of the ever shortening reporting time, and the ability to detect a wide range of organisms not limited by culturability (McClure et al., 2023; Kullar et al., 2022). A variety of molecular methods are available, with the most common being quantitative polymerase chain reaction (qPCR) and next generation sequencing (NGS). Quantitative PCR assays currently have the quickest reporting times, and can be designed to detect individual species, specific genes (e.g., antimicrobial resistance genes), or measure total bacterial or fungal load in a sample (McClure et al., 2023; Kullar et al., 2022; Fink et al., 2018; Lleo et al., 2014). Acknowledging the shortfalls in conventional testing and rapid reporting potential, some recommendations now advocate for using qPCR alongside culture in acute PJI which has shown efficacy in rapid detection for some key pathogens common in PJI (e.g., *Staphylococcus aureus*, *Streptococcus* sp.) (Ghirardelli et al., 2024). However, qPCR reports a limited set panel, identifying no more than 25–40 organisms in a test panel (Esteban et al., 2023; Gardete-Hartmann et al., 2024). In contrast, NGS methods including amplicon sequencing of the bacterial 16S ribosomal gene, are designed to detect all species in a sample that meet strict reporting criteria for NGS (Kullar et al., 2025; Indelli et al., 2025). In PJI and other orthopedic infections, NGS has shown improved sensitivity in detecting pathogenic organisms (Goswami et al., 2022; Huang et al., 2020; Flurin et al., 2022a; Azad et al., 2022) and is particularly helpful identifying organisms in culture-negative PJI (Goswami et al., 2022; Chowdhry et al., 2024). Appreciation for the clinical utility of NGS is evidenced by the advocacy for NGS in recent guidelines published by the Infectious Disease Society of America and the American Society for Microbiology (Miller et al., 2024). However, a major concern regards specificity of NGS, noting improved detection of microbes may over-diagnose PJI (Martinazzi et al., 2025). To address this concern, we compared NGS results in identifying PJI in aspirated synovial fluid samples to PJI diagnosis based on paired ICM-18 minor criteria synovial biomarkers, included in a commercially available laboratory developed test (LDT) which implements NGS (OrthoKEY, MicroGenDX, Lubbock, TX). Overall, the primary objectives of the current work were to (a) correlate broad-range 16S and ITS (i.e., NGS) positivity with biomarkers used in diagnosing PJI, (b) estimate the performance of NGS as a diagnostic for PJI, and (c) describe the bacterial and fungal composition of synovial fluid across 2,000 synovial aspirates.

## 2 Materials and methods

Following the launch of the OrthoKEY (NGS) with synovial biomarker test (components provided in Table 1), all MicroGenDX records of synovial fluid specimens submitted between December 2020 and November 2022 were flagged as eligible for retrospective study inclusion. Exclusionary criteria included specimens from sources other than the hip or knee, specimens missing data on specimen source, specimens with insufficient quantity for testing, or specimens pooled with other intraoperative sample material. Study protocol was reviewed by Advarra Center for IRB Intelligence and certified as IRB exempt (Pro#00077239). For each sample, matched NGS profiling and synovial biomarker measurements were available. Synovial biomarkers measured included c-reactive protein (CRP), white blood cell count (WBC), and polymorphonuclear neutrophil percentage (PMN%) that estimated the probability of infection, using ICM-18 minor criteria thresholds for determining elevated biomarkers (Parvizi et al., 2018). Specimens with a cumulative minor criterion score 4 or greater were considered high probability for infection, though for ICM-18, scores 4-5 are considered inconclusive and scores ≥ 6 definitive for PJI diagnosis. Limited metadata available for each patient included joint location, reported sex, and age.

**Table 1:** Components of MDX OrthoKEY plus biomarkers.

Test	Assay targets	Reports	Cutoff	ICM 2018 score
qPCR panel	Universal 16S	Bacterial cell estimate		n/a
	Common species	Estimated count/μL		n/a
	Antimicrobial resistance	Presence/absence		n/a
Targeted NGS	V1-V2 16S rRNA	Bacterial profile (%)		n/a
	ITS3-4	Fungal profile (%)		n/a
Biomarkers	CRP	mg/L CRP	>6.9 mg/L	1
	WBC	Cells/μL WBC	>3,000 cells/μL	3
	PMN%	% PMN	>80%	2

### 2.1 Synovial biomarker profiling

Synovial fluid was vortexed and 1 mL was aliquoted for biomarker analysis, with remaining synovial fluid reserved for microbial NGS processing. The synovial fluid aliquot was run on the Sysmex XN-350 (Sysmex America, Mundelein, IL) following manufacturer’s protocol quantifying WBC count and PMN from body fluids. CRP was quantified on the Pentra C400 (Horiba, Irvine, CA) platform following manufacturer’s protocol for body fluids.

### 2.2 Microbial next generation sequencing

Samples were processed via the OrthoKEY LDT (MicroGenDX, Lubbock, TX), which includes bacterial profiling via targeted amplification and sequencing of the V1-V2 regions of 16S rRNA gene, and fungal profiling based on ITS 3-4 locus sequencing as previously reported (Liss et al., 2023). Briefly, the central laboratory performs DNA extraction, PCR amplification, library preparation for sequencing on the Illumina Miseq (Illumina, Inc. San Diego, United States) platform, and bioinformatic processing of sequence data into microbial taxonomy and reporting to clinicians. Although partial 16s analysis cannot fully resolve all bacterial lineages, the underlying V1-V2 region used here is among the most informative for classifying to the species level (Kullar et al., 2025). Further, species-level identification is generally reproducible when using appropriately curated reference databases (Hoffman et al., 2021). Microbial taxa are aggregated and reported to the species level where possible, as reported in real time to clinicians and similar to prior work (Goswami et al., 2022; Liss et al., 2023; Whitfield et al., 2024).

The laboratory protocol includes a standardized approach for determining whether a sample is NGS positive or negative, intended to reduce false positive reporting and focus on dominant organisms detected as previously described (Kullar et al., 2025). To be reported positive, a sample must first pass through broad-range PCR amplification of the partial 16s rRNA (bacterial) and ITS (fungal) genes using 35 cycles and producing an amplicon product that can be visualized by gel electrophoresis (i.e., end point PCR). Samples with a positive product are barcoded and prepared for sequencing. To improve sensitivity, samples failing to produce a band on first attempt were re-evaluated further at 3X higher input volume. Samples fail if a band is not produced after second PCR attempt. After sequencing, samples are evaluated for contamination by a proprietary process comparing specimens to negative extraction controls (extraction without patient specimen) and no template PCR controls (PCR amplification without DNA extract), and positive controls. Contaminant signals are removed from results along with any species detected under 2% relative proportion of each sample’s total read count. Samples may fail if the Quality Control (QC) process flags a high enough amount of a sample’s composition as contamination. The determination of a contaminant signal is based on mutual detection rates and abundance values between patient samples and concurrently run negative controls. This LDT is currently in good standing with the College of American Pathologists (CAP) accreditation, Clinical Laboratory Improvement Amendments (CLIA) licensing, and New York state’s Clinical Laboratory Evaluation Program (CLEP).

### 2.3 Statistical analysis

Statistical analysis was performed using R programming environment. Tables were generated using kable or gtsummary (Sjoberg et al., 2021) libraries. Descriptive analyses were first performed to report on NGS positivity and ICM-18 minor criteria infected rates across hips and knee samples, as well as distributions of samples across interactions of positive NGS with positive ICM criteria. A kappa test was used to evaluate the agreement between NGS positivity and PJI. To compare diagnostic performance of individual synovial markers against PJI, the “epi.test” R function (epiR library) was used to estimate sensitivity, specificity, accuracy, positive predictive value (PPV), and negative predictive value (NPV). Wilson’s 95% confidence limits were estimated for each test value.

## 3 Results

**Figure 1:**
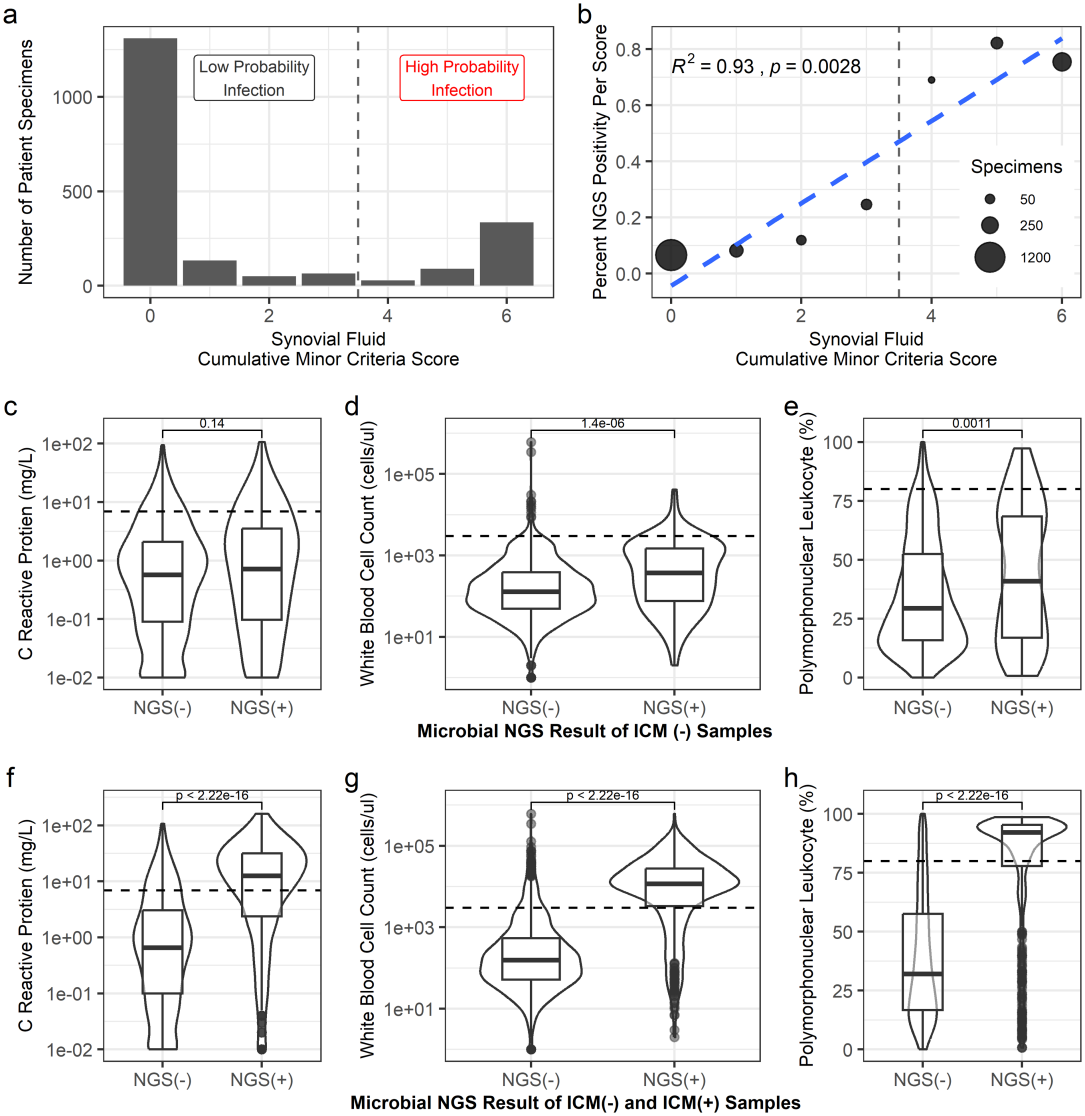
Comparisons of NGS positivity to ICM synovial biomarkers. **(a)** Histogram shows the distribution of specimens across each possible ICM score. A vertical dashed line indicates the threshold used in the current study for high probability infection adapted from the 2018 ICM minor criteria. **(b)** The percentage of NGS positive samples was calculated for each possible ICM score. A direct linear relationship was observed between cumulative ICM scores and aggregated NGS positivity. **(c–h)** Box plots show the relationship between NGS positivity and raw biomarker score distributions for **(c,f)** CRP, **(d,g)** WBC count, and **(e,h)** PMN%. For the first set **(c–e)**, NGS positivity was compared to biomarkers in uninfected samples whereas **(f–h)** compares biomarker values in all samples by NGS positivity. In the box plots, box indicates the 1st and 3rd quartiles, the thicker banded line within the box indicates the median, and the whiskers indicate the bounds of the distribution. A violin plot layer visualizes the full data distribution. A two-tailed *t*-test was used to determine statistical significance by NGS positivity and each biomarker, with *p*-value shown in plot. A dashed horizontal line indicates ICM thresholds for elevated biomarkers.

A total of 2011 synovial fluid specimens were included with matched NGS profiling and biomarkers (Table 2). A greater proportion of specimens originated from the knee (86%) compared to the hip (14%). There was no difference in the distribution of patients by sex or age between the two joints (*p* > 0.05). The median time to report results was 4 days (Q1 = 3, Q3 = 5) from the time samples were received at the central lab. Overall, the observed NGS positivity was 23% in both hips and knees, whereas 24% of hips and 22% of knees met the ICM-18 minor criteria for high probability PJI (Table 3, Figure 1a). Of positive results, 448 were 16S bacterial and 26 ITS fungal positive results.

**Table 2:** Patient demographics and primary results by joint.

Characteristic	Hip, *N* = 287	Knee, *N* = 1,724
Mean age (SD)	65 (11)	67 (11)
Sex (male)	146 (51%)	931 (54%)
NGS positivity	66 (23%)	401 (23%)
PJI (high probability)	68 (24%)	386 (22%)

**Table 3:** High/low probabilities per NGS positivity in each joint.

Result per assay	Knee		Hip	
	High PJI probability *N* = 386 (22%)	Low PJI probability *N* = 1,338 (78%)	High PJI probability *N* = 68 (24%)	Low PJI probability *N* = 219 (76%)
NGS result overall				
NEG	88 (23%)	1,235 (92%)	19 (28%)	202 (92%)
POS	298 (77%)	103 (7.7%)	49 (72%)	17 (7.8%)
16s				
NEG	94 (24%)	1,247 (93%)	19 (28%)	203 (93%)
POS	292 (76%)	91 (6.8%)	49 (72%)	16 (7.3%)
ITS				
NEG	376 (97%)	1,323 (99%)	68 (100%)	218 (100%)
POS	10 (2.6%)	15 (1.1%)	0 (0%)	1 (0.5%)

### 3.1 Broad-range NGS positivity comparisons to synovial biomarkers

For most of the analysis presented herein, the synovial biomarker results were analyzed according to their pooled finding of low or high probability infection for PJI following ICM-18. However, a brief analysis also investigated if NGS positivity was correlated with the ICM minor criteria score, noting a score ≥4 was considered high probability for infection. A highly significant linear relationship was observed between aggregated NGS positivity rate and cumulative biomarker score (*p* = 0.0028, *R*^2^ = 0.93, Figure 1b). For example; Within ICM-negative samples, samples with an ICM score of zero had the lowest NGS positivity rate of 6.6% which increased to 24.6% at an ICM score of 3. In these low probability of infection samples, NGS positivity was found to be significantly associated with both elevated WBC (*p* < 0.0001) and PMN values (*p* = 0.0011), but not CRP (*p* = 0.14, Figures 1c–e).

NGS positivity was next compared to the three measured synovial biomarkers to evaluate the concordance to raw lab values of all samples. In each case, NGS positivity was found to be associated with increased values (*p* < 0.001; Figures 1f–h), particularly WBC and PMN%. Though NGS positivity was still largely discriminatory for CRP, the effect size is smaller and there is relatively more overlap. This led to the question of whether synovial NGS positivity was more concordant with PJI than synovial CRP. Indeed, the specificity of NGS positivity was observed at 92.3% (95% CI: 0.909–0.935, Table 4), greater than CRP at 89.4% (0.878–0.908) but lower than WBC at 97.9% (0.97–0.985) and PMN% at 94.7% (0.935–0.957).

**Table 4:** Comparing diagnostic performance of individual synovial fluid test results to ICM-18 minor criteria for overall diagnosis by synovial markers.

Synovial diagnostic	Sensitivity	Specificity	Accuracy	PPV	NPV
CRP	0.802 (0.763–0.836)	0.894 (0.878–0.908)	0.873 (0.858–0.887)	0.688 (0.647–0.726)	0.939 (0.926–0.95)
WBC	1 (0.992–1)	0.979 (0.97–0.985)	0.984 (0.977–0.988)	0.932 (0.906–0.951)	1 (0.997–1)
PMN (%)	0.936 (0.91–0.955)	0.947 (0.935–0.957)	0.945 (0.934–0.954)	0.838 (0.804–0.868)	0.981 (0.972–0.987)
NGS positivity	0.764 (0.723–0.801)	0.923 (0.909–0.935)	0.887 (0.873–0.9)	0.743 (0.702–0.781)	0.931 (0.917–0.942)

PPV, positive predictive value; NPV, negative predictive value.

### 3.2 NGS positivity is concordant with high probability PJI

NGS positivity among ICM positive specimens was 77% in knees and 72% in hips with 7.7 and 7.8% potential false positives, respectively (Table 3). Kappa testing was used to evaluate the chance corrected agreement between NGS and PJI. Overall, NGS positivity was substantially concordant (kappa = 0.68) and 88.71% accurate (95% CI 87.25–90.06) to PJI (Table 5). Concordance for hips and knees were each very similar to the overall findings, though with a higher sensitivity for knees (77.2%) as compared to hips (72.06%). Much of the observed sensitivity was due to the bacterial 16S rather than fungal ITS. For ICM positive knees, bacterial 16S and fungal ITS positivity were 76 and 2.6%, respectively. ICM positive hips had lower detection rates at 72% 16S positivity and 0% ITS positivity (Table 3).

**Table 5:** Concordance of broad-range NGS positivity with biomarkers overall, and within each joint.

Joint	*N*	Accuracy	95% CI	Sensitivity	Specificity	PPV	NPV	Kappa
Overall	2011	88.71%	87.25%–90.06%	76.43%	92.29%	74.3%	93.07%	0.6803
Knee	1,724	88.92%	87.34%–90.36%	77.2%	92.3%	74.31%	93.35%	0.6856
Hip	287	87.46%	83.06%–91.06%	72.06%	92.24%	74.24%	91.4%	0.6495

### 3.3 Microbial NGS profile

**Figure 2:**
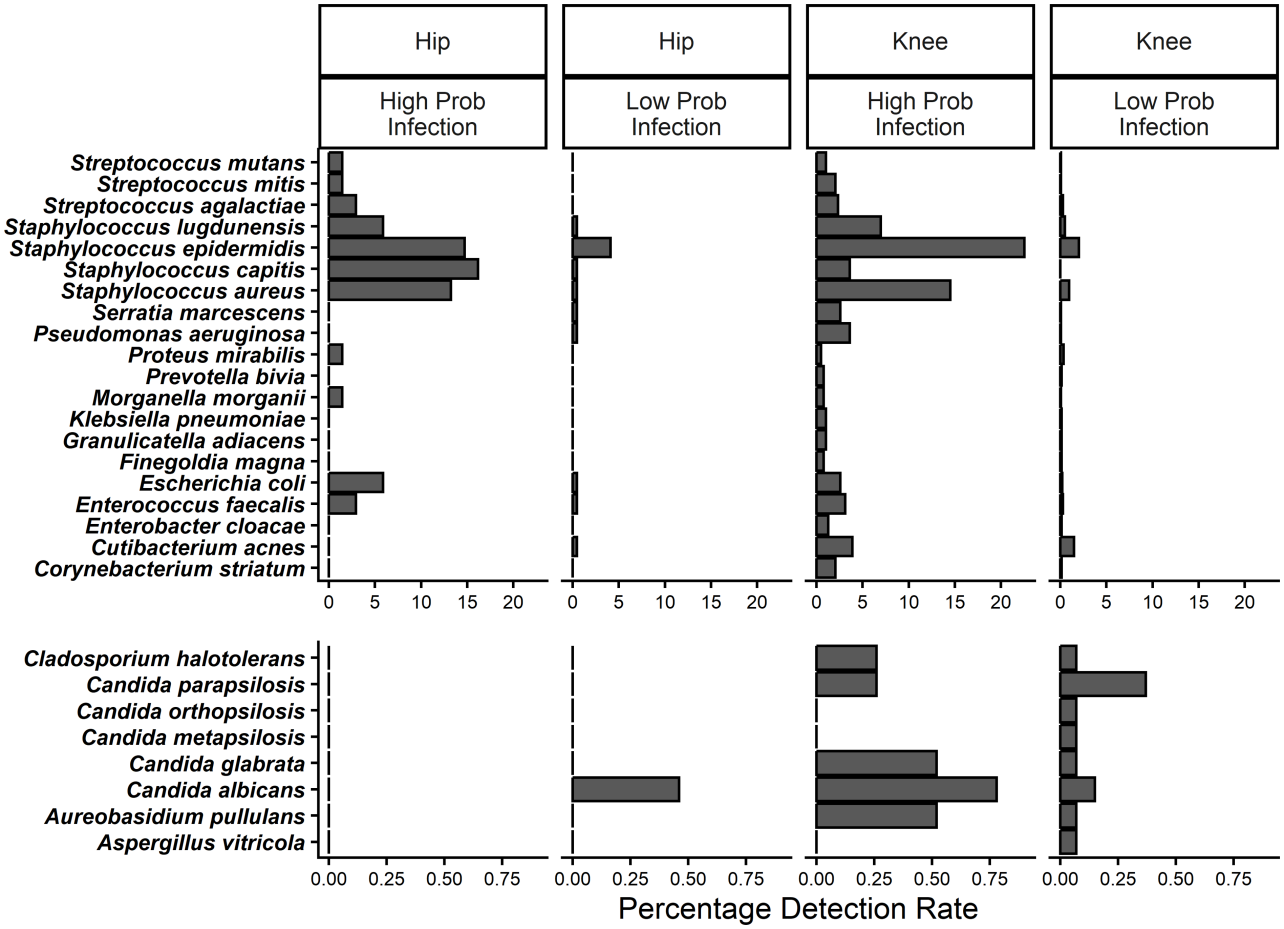
Comparing the percentage detection rate by setting and probability of infection across top species by incidence.

A total of 160 unique bacterial species were detected with 69 being the dominant organism (>50% relative abundance) in at least one sample versus 16 unique fungal species and 13 dominant fungi. There was a median 1 species reported per NGS positive sample and 104 (22.3%) were polymicrobial. Staphylococcal species were the most frequent dominant species, reported in 51% of samples and including *S. epidermidis*, *S. aureus*, *S. lugdunensis*, and *S. capitis*. Additional species with more than 2% dominance included *Streptococcus agalactiae* (2.9%), *Pseudomonas aeruginosa* (2.9%), *Cutibacterium acnes* (4.0%) and *Enterococcus faecalis* (2.9%). These eight species composed 64% of the dominant species reported with 61 relatively less common species being dominant in the remaining 160 specimens (36%). Fungal detections were generally rare, observed in 2.2% (Mortazavi et al., 2011) of all PJI specimens and in 1.0% (Esteban et al., 2023) of PJI-negative specimens. Species included several *Candida* species (*C. albicans*, *C. glabrata*, *C. parapsilosis*, etc. …), *Aureobasidium pullulans*, *Malassezia globosa*, and *Naganishia diffluens* (Figure 2, Supplementary Table 2).

**Figure 3:**
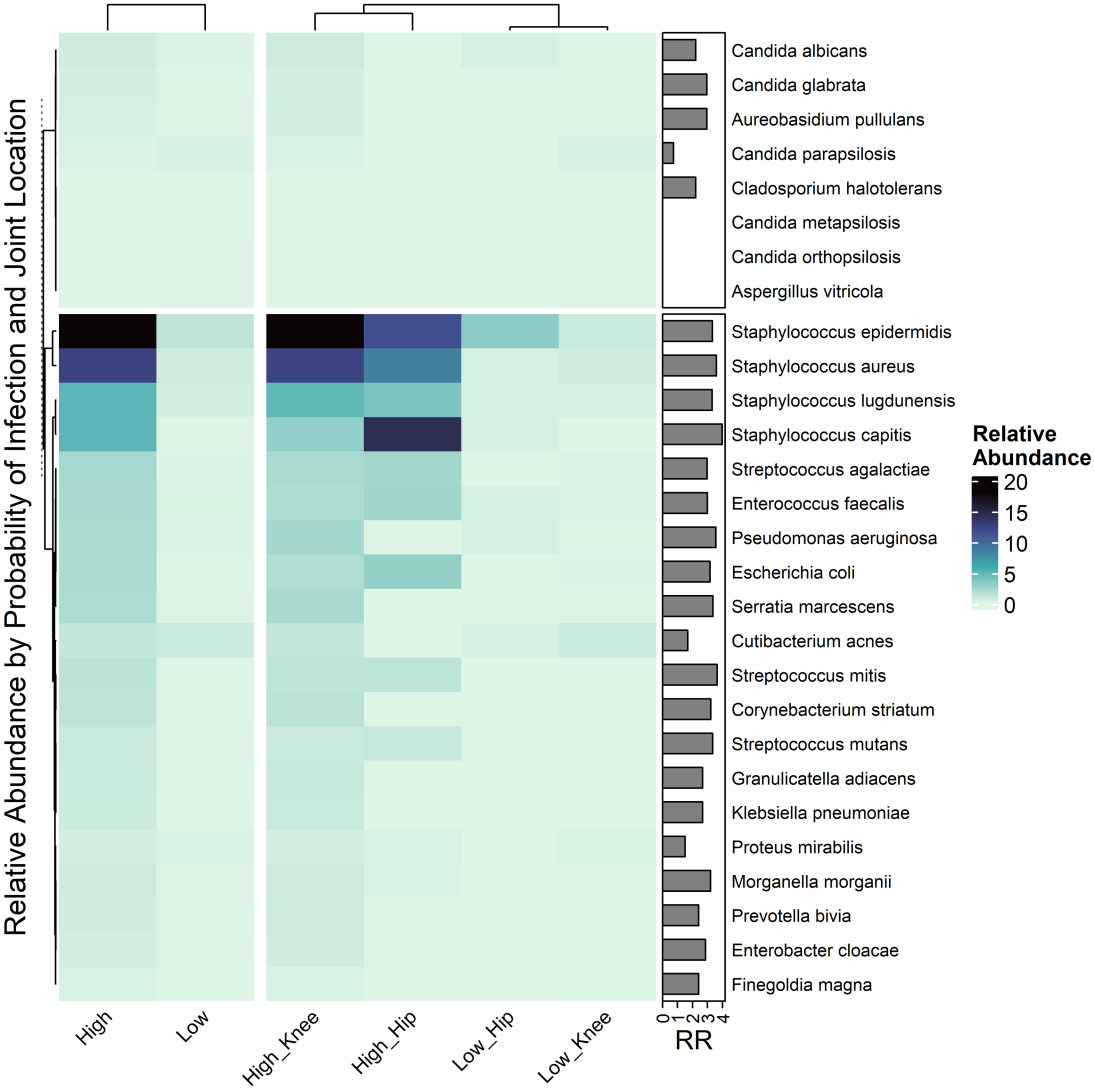
Mean relative abundance by joint location and infection probability. Right annotation references the Relative Risk of Higher probability of infection per species. Values over one, signifies an increased risk of having a higher probabilty of infection with exposure to species.

Consistent with the high rate of NGS positivity associated with high probability infection samples, incidence and mean relative abundance of the most common species heavily favored specimens scored as high probability infection (Figures 2, 3). A few of these top species were noted to be generally consistent with hips and knees such as *S. epidermidis* and *S. aureus*, however others can be seen to appear more distinct between the two joints. For example, Figure 3 separates species according to their joint-wise distributions and notes a few lineages including *S. capitis* and *Streptococcus parasanguinis*, among others, more abundant in hips. *Cutibacterium acnes* was a notable departure from all other species in that *C. acnes* appeared less discriminatory by infection probability. Logistic regression was used to estimate the relative risk of PJI based on each species, whereas *C. acnes* detection still indicated more than 2X greater relative risk for PJI, this risk was lower than for other species detected as abundantly (Figure 3). Of the fungal species detected, *Candida albicans*, *C. glabrata*, and *A. pullulans* were generally detected at higher rates in PJI samples and dominated the fungal composition of at least two samples, whereas other *Candida* species (*C. parapsilosis*, *C. orthopsilosis*, *C. metapsilosis*) were more frequent and dominant among PJI-negative specimens (Figure 2, Supplementary Table 2).

## 4 Discussion

This study was conducted to benchmark the performance of a commercially available NGS technology compared to synovial fluid biomarkers used to diagnose PJI using ICM-18 criterion. We hypothesized that positive DNA signals in synovial fluid signifies the presence of clinically relevant microbes, and that positive NGS detection will associate with biomarkers indicative of infection. This study shows NGS positivity is concordant with diagnostic PJI ICM minor criteria biomarkers, evidenced by the substantial agreement observed by kappa testing and performance statistics, and had greater specificity than CRP (Tables 3, 4). Consistent with prior literature (Kuo et al., 2022), WBC and PMN individually showed generally superior performance in all measured parameters to CRP as well as NGS for diagnosis of PJI (Table 4). Further, NGS positivity was associated with elevated levels for each tested biomarker (Figure 1). These comparisons to established biomarkers for diagnosing PJI establish a clinical correlation between NGS broad-range positivity and validated markers for infection. The lower overall sensitivity and accuracy of NGS compared to WBC and PMN suggest that NGS would not replace WBC or PMN, but NGS may fill a complementary role similar to fluid culture that can aid in cases where the diagnosis is unclear by traditional markers and provide a greater chance at capturing causative microbes that may be missed by conventional microbiology testing prior to surgery.

One concern with incorporating NGS as a standard of care for PJI diagnosis is that increased sensitivity will lead to a high false positive rate and unnecessary treatment (McClure et al., 2023). This study refutes this concern. NGS positivity demonstrated a comparable and non-inferior specificity to prior estimates at 92.3% (95% CI: 0.91–0.94, Tables 4, 5). This is supported by a recent meta-analysis including 18 studies examining the diagnostic performance of synovial fluid cultures against PJI which observed 96% specificity (95% CI: 0.93–0.98) (Watanabe et al., 2024). Moreover, we report a superior increase in sensitivity for NGS at 76.4% (95% CI: 0.72–0.80) compared to the prior pooled sensitivity for culture at 63% (95% CI: 0.56–0.70). While we intend future studies with matched culture and NGS results to corroborate this finding, the present data show that NGS, using synovial fluid singularly, is both significantly more sensitive than prior expectations for culture and comparably specific to other validated biomarkers for diagnosing PJI.

The comparisons of NGS positivity to biomarkers in samples deemed low probability of infection emphasizes the complementary role that NGS can provide in diagnosing PJI (Figures 1b–e). First, a highly significant relationship was observed between NGS positivity and cumulative ICM score, suggesting that samples scored as a three with 1-2 elevated biomarkers also show higher NGS positivity rates when compared to other ICM negative samples. Further, NGS positivity was highly significantly related to both elevated PMN and WBC when considering only ICM negative samples. Synovial WBC and PMN are considered among the most reliable synovial biomarkers for PJI (Kuo et al., 2022; McNally et al., 2021). These findings support that NGS based detection of microbial DNA in synovial fluid may be capturing a unique component of PJI that is missed by only assessing the more commonly measured biomarkers. More specifically, we hypothesize that some of these discrepant NGS positive results may represent infection from microbes failing to elicit a strong immune response, a sub-clinical infection, or perhaps early detection of an infection that has not yet fully manifested clinically. Future work examining longitudinal trends in microbiological findings, synovial biomarkers, and clinical findings will be critical in further understanding the value of these discrepant results. Nevertheless, the strong relationship of PMN and WBC with NGS positivity in low probability infection samples supports the potential for NGS positivity as a diagnostic criterion to improve sensitivity in diagnosing PJI, especially in difficult cases involving fastidious microbes associated with ambiguous biomarker values according to ICM-18 definitions.

In addition to the role in diagnosing PJI as well as identifying microbes in culture-negative infection (Goswami et al., 2022; Kullar et al., 2022), NGS is capable of detecting a wide variety of organisms which may be missed by culture-dependent identification as well as other molecular methods limited by species specific probes or antibodies (Chowdhry et al., 2024). Consistent with prior literature, we report a high prevalence of *Staphyloccocus aureus*, coagulase negative *Staphylococcus* (*S. epidermidis*, *S. capitis*), *S. lugdunensis*, *Streptococcus agalactiae* (i.e., GBS), *Pseudomonas*, and *Cutibacterium acnes*. Organisms underreported by culture-dependent methods were also observed, often as the dominant organism, particularly anaerobes such *Anaerococcus*, *Bacteroides*, and *Finegoldia magna* (Figures 2, 3). Similarly, *Cutibacterium acnes* is underreported compared to molecular methods (Fink et al., 2018; Morgenstern et al., 2018). Eight bacterial species dominated the composition in 68% of samples, though the remaining 32% consisted of 46 different dominant species (Supplementary Table 1). This result highlights that although any given species such as *Enterococcus faecium* or *Streptococcus dysgalactiae* may be individually uncommon, uncommon species are a common aspect in PJI which may be overlooked by routine microbiological testing. For example, the broader detection range enabled by NGS is more informative than the commonly used qPCR BioFire Joint Infection Panel (bioMerieux, Marcy-l’Etoile, FR) which, in comparison, could only detect 56% of the 69 dominant species identified in this current work. While such panels are useful in being able to deliver rapid results of common pathogens and there is a growing role for use in acute PJI (comprehensively reviewed here 19), the limited qPCR panels have shown lower overall sensitivity to PJI ranging 41–56% (Gardete-Hartmann et al., 2024; Azad et al., 2022; Lee, 2024) and are known to underreport polymicrobial instances of PJI (Azad et al., 2022).

Of the more prevalent organisms reported by NGS, *C. acnes* was uniquely abundant overall and reported more frequently in ICM low probability infection samples according to minor criteria (Figure 2). This finding was not unexpected, as *C. acnes* is frequently reported as a causative pathogen in PJI (Hedlundh et al., 2021), yet it has also been shown to elicit a variable response from the host, relegating its positivity as a potential contaminant (Hoch et al., 2023). Reliable detection of *C. acnes* via culture can be a challenge requiring anaerobic isolation and extended 14-day incubation time (Renz et al., 2018; Nodzo et al., 2017). Further, *C. acnes* does not always elicit a strong host response and has been associated with PJI manifesting later in the implant life cycle (Renz et al., 2018; Liew-Littorin et al., 2024; Vilchez et al., 2021). Thus, early confirmation via NGS is useful, providing impetus toward treatment rather than neglecting equivocal synovial biomarker data in patients with a painful JA. When detected, *C. acnes* JA infections have high treatment success and 2-year survival rates (Hedlundh et al., 2021; Hoch et al., 2023; Warne et al., 2024), indicating that *C. acnes* associated PJI is manageable if detected. Further, current results may support treating NGS detected *C. acnes* in circumstances of ICM lower probability of PJI, acknowledging further study is required in understand treating *C. acnes* when isolated in the setting of a diminished inflammatory response (Nodzo et al., 2017; Liew-Littorin et al., 2024).

Although many studies contrast the utility of culture and NGS, we believe that consensus guidelines will benefit by considering information from both techniques, rather than one or the other, noting improved sensitivity of microbiological testing for PJI when both are used (Flurin et al., 2022b). A recent comprehensive review of NGS in PJI reports strong sensitivity and specificity (Martinazzi et al., 2025). Consistent with a recent comprehensive review of molecular testing for PJI (Indelli et al., 2025), we advocate the adoption of NGS, especially in culture-negative cases, when rapid pathogen identification is needed, in patients with a high-pretest probability of infection, or when rare pathogens are suspected. We emphasize this study shows rare pathogens to be collectively common, including microbes not included in common multiplex qPCR panels and fastidious organisms likely to be missed by culture. There is concern that NGS may lead to increased false positive detection, but the specificities shown here and the accuracies reported in the review by Martinazzi et al. (2025) refute that concern. We recommend that NGS as implemented herein is a useful tool that will assist with identifying PJI causative organisms, confirming PJI diagnosis, and should be considered as a criterion in PJI diagnosis.

The current study supports the use of NGS in guiding PJI diagnosis, however not without limitations. We only compared NGS to biomarkers of ICM-18 minor criteria which did not include culture, information on other ICM criteria for infection, nor other information from the patients’ medical records which may have been helpful assessing the value of additional NGS information. However, when using synovial biomarkers, NGS showed superior sensitivity to prior expectations for culture and non-inferior or comparable specificity to currently accepted indicators for infection. While additional data could have helped to define PJI, there is a reported 88% concordance between minor and major criteria in diagnosing PJI (Klement et al., 2018). Also, NGS results were compared only to synovial fluid biomarkers, absent alpha defensin, and not to ICM-18 serum biomarkers that include CRP, D-dimer, and sedimentation rate. At the time of writing, a single-center follow up study is underway comparing matched synovial fluid aspirations sent for matched NGS, culture, and additional data points. As the current study was based solely on records collected through the central laboratory, we did not have access to information such as antibiotics the patients may have been on at time of sampling or prescribed after, nor information on how treatment or clinical outcomes were affected. We intend future studies to dive further into these impacts for NGS use in fluid aspirations. Information on precisely how treatment and outcomes are affected would be especially valued in determining the utility of culture-negative PJI cases with positive NGS or cases determined to be polymicrobial through NGS. The current work did not consider intraoperative targeted NGS results, which has shown superior sensitivity in microbial detection for PJI up to 89.3% by the same comparator lab (Tarabichi et al., 2018). We emphasize the focus of this study is synovial fluid analysis for preoperative diagnosis of PJI. A positive diagnosis provides surgeon clarity and a direction of treatment before surgery, rather than modifying post-operative treatment when the joint of interest is determined PJI positive based on intra-operative ICM-18 criteria.

Developing workflows that incorporate NGS into standard practice raises questions about accessibility and cost effectiveness. Underscoring cost concerns, a survey by the Infectious Disease Society of America (IDSA) indicated that 33% of infectious disease physicians had been prevented from using molecular diagnostic testing by cost and payor concerns despite 78% of respondents answering that molecular testing was occasionally or often helpful in clinical decision making (Rao et al., 2024). At time of writing, the test evaluated by the current paper is typically priced at $400/test and recently available in European markets at ~€400. Torchia et al. (2019) previously concluded in a cost analysis that NGS could be cost effective up to $3,916 per test assuming the test was at least 71% sensitive and 94% specific to PJI. More comprehensive follow up study will be useful to understand cost effectiveness, but the currently evaluated test is well below the proposed cost effectiveness point. Following cost, lack of cohesive guidelines for using molecular testing was the next most cited barrier preventing or creating difficulty for the use of molecular testing (Rao et al., 2024). Given the recent conclusion of the ICM2025 meeting and upcoming joint European Bone and Joint Infection Society (EBJIS)/MusculoSkeletal Infection Society (MSIS) meeting, the current work is expected to contribute additional perspective on the value of NGS for preoperative diagnosis as working groups convene to improve criteria for PJI.

We believe a positive NGS test is commensurate with existing ICM-18 minor criteria diagnosing PJI. Based on this data, we feel that NGS testing may have a role as a primary diagnostic tool, performed early as opposed to a secondary diagnostic when other testing has already failed. If considered as a preoperative diagnostic tool, we suggest a positive NGS be weighted similar to a positive culture when restructuring future PJI diagnostic criterion. Further, we believe NGS will close the gap in culture negative infection as NGS enables detection of fastidious microbes thus allowing fidelity in antimicrobial stewardship. The enhanced sensitivity for microbial detection can be used to prepare targeted treatment of PJI causative organisms. We look forward to seeing and conducting further research on the implementation of NGS into the management of PJI.

## 5 Conclusion

Microbial NGS testing of synovial fluid showed concordance to biomarkers used in ICM-18 PJI minor criteria. NGS showed a low risk of false positive detections in synovial fluid. Further work in matched samples with matched culture and NGS synovial fluid profiling is warranted to understand the potential margin of benefit, but current results suggest NGS offers broad discovery power for pathogens, is specific to PJI, and may warrant guidance as a diagnostic criterion when restructuring future diagnostic criteria defining PJI.

## Data Availability

The original contributions presented in the study are included in the article/Supplementary material, further inquiries can be directed to the corresponding author/s.
