# Supplementary material for: Microbial next generation DNA sequencing of aspirated synovial fluid shows concordance with ICM criteria biomarkers for diagnosing periprosthetic joint infection in hip and knee arthroplasty
Source: Front Microbiol. 2026 May 11;17:1816780. doi: 10.3389/fmicb.2026.1816780 (PMC13199172; doi:10.3389/fmicb.2026.1816780)
Supplement: Supplementary file 1 [file Table_1.docx]

Table S1. Dominant bacterial species detected per high and low probability infection, per hip or knee

| Bacteria | Overall N=448 | High Hip N=49 | High Knee N=292 | Low Hip N=16 | Low Knee N=91 |
| --- | --- | --- | --- | --- | --- |
| *Staphylococcus epidermidis* | 107 (24%) | 9 (18%) | 78 (27%) | 9 (56%) | 11 (12%) |
| *Staphylococcus aureus* | 69 (15%) | 6 (12%) | 52 (18%) | 1 (6.3%) | 10 (11%) |
| *Staphylococcus lugdunensis* | 30 (6.7%) | 3 (6.1%) | 20 (6.8%) | 1 (6.3%) | 6 (6.6%) |
| *Staphylococcus capitis* | 25 (5.6%) | 11 (22%) | 13 (4.5%) | 1 (6.3%) | 0 (0%) |
| *Cutibacterium acnes* | 18 (4.0%) | 0 (0%) | 4 (1.4%) | 1 (6.3%) | 13 (14%) |
| *Enterococcus faecalis* | 13 (2.9%) | 2 (4.1%) | 8 (2.7%) | 1 (6.3%) | 2 (2.2%) |
| *Pseudomonas aeruginosa* | 13 (2.9%) | 0 (0%) | 11 (3.8%) | 1 (6.3%) | 1 (1.1%) |
| *Streptococcus agalactiae* | 13 (2.9%) | 2 (4.1%) | 9 (3.1%) | 0 (0%) | 2 (2.2%) |
| *Escherichia coli* | 12 (2.7%) | 3 (6.1%) | 8 (2.7%) | 0 (0%) | 1 (1.1%) |
| *Serratia marcescens* | 10 (2.2%) | 0 (0%) | 9 (3.1%) | 0 (0%) | 1 (1.1%) |
| *Corynebacterium striatum* | 8 (1.8%) | 0 (0%) | 7 (2.4%) | 0 (0%) | 1 (1.1%) |
| *Streptococcus mitis* | 8 (1.8%) | 1 (2.0%) | 6 (2.1%) | 0 (0%) | 1 (1.1%) |
| *Proteus mirabilis* | 6 (1.3%) | 0 (0%) | 2 (0.7%) | 0 (0%) | 4 (4.4%) |
| *Streptococcus mutans* | 6 (1.3%) | 1 (2.0%) | 4 (1.4%) | 0 (0%) | 1 (1.1%) |
| *Granulicatella adiacens* | 5 (1.1%) | 0 (0%) | 4 (1.4%) | 0 (0%) | 1 (1.1%) |
| *Enterobacter cloacae* | 4 (0.9%) | 0 (0%) | 3 (1.0%) | 0 (0%) | 1 (1.1%) |
| *Klebsiella pneumoniae* | 4 (0.9%) | 0 (0%) | 4 (1.4%) | 0 (0%) | 0 (0%) |
| *Morganella morganii* | 4 (0.9%) | 0 (0%) | 3 (1.0%) | 0 (0%) | 1 (1.1%) |
| *Prevotella bivia* | 4 (0.9%) | 0 (0%) | 3 (1.0%) | 0 (0%) | 1 (1.1%) |
| *Streptococcus gordonii* | 3 (0.7%) | 0 (0%) | 3 (1.0%) | 0 (0%) | 0 (0%) |
| *Streptococcus parasanguinis* | 3 (0.7%) | 2 (4.1%) | 1 (0.3%) | 0 (0%) | 0 (0%) |
| *Veillonella parvula* | 3 (0.7%) | 0 (0%) | 2 (0.7%) | 0 (0%) | 1 (1.1%) |
| *Acinetobacter johnsonii* | 2 (0.4%) | 0 (0%) | 0 (0%) | 1 (6.3%) | 1 (1.1%) |
| *Capnocytophaga canimorsus* | 2 (0.4%) | 0 (0%) | 2 (0.7%) | 0 (0%) | 0 (0%) |
| *Enterobacter sp* | 2 (0.4%) | 1 (2.0%) | 1 (0.3%) | 0 (0%) | 0 (0%) |
| *Enterococcus faecium* | 2 (0.4%) | 1 (2.0%) | 1 (0.3%) | 0 (0%) | 0 (0%) |
| *Finegoldia magna* | 2 (0.4%) | 0 (0%) | 1 (0.3%) | 0 (0%) | 1 (1.1%) |
| *Klebsiella aerogenes* | 2 (0.4%) | 1 (2.0%) | 0 (0%) | 0 (0%) | 1 (1.1%) |
| *Staphylococcus haemolyticus* | 2 (0.4%) | 0 (0%) | 1 (0.3%) | 0 (0%) | 1 (1.1%) |
| *Staphylococcus hominis* | 2 (0.4%) | 0 (0%) | 0 (0%) | 0 (0%) | 2 (2.2%) |
| *Streptococcus dysgalactiae* | 2 (0.4%) | 0 (0%) | 2 (0.7%) | 0 (0%) | 0 (0%) |
| *Streptococcus oralis* | 2 (0.4%) | 0 (0%) | 2 (0.7%) | 0 (0%) | 0 (0%) |
| *Acinetobacter lwoffii* | 1 (0.2%) | 0 (0%) | 0 (0%) | 0 (0%) | 1 (1.1%) |
| *Bacillus sp* | 1 (0.2%) | 0 (0%) | 0 (0%) | 0 (0%) | 1 (1.1%) |
| *Bacteriodes fragilis* | 1 (0.2%) | 0 (0%) | 1 (0.3%) | 0 (0%) | 0 (0%) |
| *Campylobacter jejuni* | 1 (0.2%) | 1 (2.0%) | 0 (0%) | 0 (0%) | 0 (0%) |
| *Campylobacter ureolyticus* | 1 (0.2%) | 1 (2.0%) | 0 (0%) | 0 (0%) | 0 (0%) |
| *Candidatus-Burkholderia crenata* | 1 (0.2%) | 0 (0%) | 0 (0%) | 0 (0%) | 1 (1.1%) |
| *Citrobacter koseri* | 1 (0.2%) | 0 (0%) | 1 (0.3%) | 0 (0%) | 0 (0%) |
| *Corynebacterium amycolatum* | 1 (0.2%) | 0 (0%) | 0 (0%) | 0 (0%) | 1 (1.1%) |
| *Corynebacterium sp* | 1 (0.2%) | 0 (0%) | 0 (0%) | 0 (0%) | 1 (1.1%) |
| *Cutibacterium avidum* | 1 (0.2%) | 1 (2.0%) | 0 (0%) | 0 (0%) | 0 (0%) |
| *Exiguobacterium acetylicum* | 1 (0.2%) | 0 (0%) | 0 (0%) | 0 (0%) | 1 (1.1%) |
| *Exiguobacterium undae* | 1 (0.2%) | 0 (0%) | 0 (0%) | 0 (0%) | 1 (1.1%) |
| *Gemella haemolysans* | 1 (0.2%) | 0 (0%) | 1 (0.3%) | 0 (0%) | 0 (0%) |
| *Gemella morbillorum* | 1 (0.2%) | 1 (2.0%) | 0 (0%) | 0 (0%) | 0 (0%) |
| *Haemophilus parainfluenzae* | 1 (0.2%) | 0 (0%) | 1 (0.3%) | 0 (0%) | 0 (0%) |
| *Kocuria marina* | 1 (0.2%) | 0 (0%) | 0 (0%) | 0 (0%) | 1 (1.1%) |
| *Lactobacillus gasseri* | 1 (0.2%) | 0 (0%) | 1 (0.3%) | 0 (0%) | 0 (0%) |
| *Lactococcus lactis* | 1 (0.2%) | 0 (0%) | 1 (0.3%) | 0 (0%) | 0 (0%) |
| *Lysinibacillus massiliensis* | 1 (0.2%) | 0 (0%) | 1 (0.3%) | 0 (0%) | 0 (0%) |
| *Megamonas funiformis* | 1 (0.2%) | 0 (0%) | 0 (0%) | 0 (0%) | 1 (1.1%) |
| *Methylobacterium populi* | 1 (0.2%) | 0 (0%) | 1 (0.3%) | 0 (0%) | 0 (0%) |
| *Micrococcus luteus* | 1 (0.2%) | 0 (0%) | 0 (0%) | 0 (0%) | 1 (1.1%) |
| *Neisseria mucosa* | 1 (0.2%) | 0 (0%) | 0 (0%) | 0 (0%) | 1 (1.1%) |
| *Pasteurella dagmatis* | 1 (0.2%) | 0 (0%) | 1 (0.3%) | 0 (0%) | 0 (0%) |
| *Prevotella melaninogenica* | 1 (0.2%) | 0 (0%) | 1 (0.3%) | 0 (0%) | 0 (0%) |
| *Propionibacterium namnetense* | 1 (0.2%) | 0 (0%) | 0 (0%) | 0 (0%) | 1 (1.1%) |
| *Rhodotorula sp* | 1 (0.2%) | 0 (0%) | 1 (0.3%) | 0 (0%) | 0 (0%) |
| *Staphylococcus pettenkoferi* | 1 (0.2%) | 0 (0%) | 1 (0.3%) | 0 (0%) | 0 (0%) |
| *Staphylococcus pseudintermedius* | 1 (0.2%) | 0 (0%) | 1 (0.3%) | 0 (0%) | 0 (0%) |
| *Staphylococcus saprophyticus* | 1 (0.2%) | 0 (0%) | 1 (0.3%) | 0 (0%) | 0 (0%) |
| *Streptococcus anginosus* | 1 (0.2%) | 0 (0%) | 1 (0.3%) | 0 (0%) | 0 (0%) |
| *Streptococcus canis* | 1 (0.2%) | 0 (0%) | 1 (0.3%) | 0 (0%) | 0 (0%) |
| *Streptococcus equinus* | 1 (0.2%) | 0 (0%) | 1 (0.3%) | 0 (0%) | 0 (0%) |
| *Streptococcus pyogenes* | 1 (0.2%) | 1 (2.0%) | 0 (0%) | 0 (0%) | 0 (0%) |
| *Streptococcus salivarius* | 1 (0.2%) | 0 (0%) | 1 (0.3%) | 0 (0%) | 0 (0%) |
| *Streptococcus sp* | 1 (0.2%) | 1 (2.0%) | 0 (0%) | 0 (0%) | 0 (0%) |
| *Streptococcus vestibularis* | 1 (0.2%) | 0 (0%) | 1 (0.3%) | 0 (0%) | 0 (0%) |
