# Supplementary material for: Microbial next generation DNA sequencing of aspirated synovial fluid shows concordance with ICM criteria biomarkers for diagnosing periprosthetic joint infection in hip and knee arthroplasty
Source: Front Microbiol. 2026 May 11;17:1816780. doi: 10.3389/fmicb.2026.1816780 (PMC13199172; doi:10.3389/fmicb.2026.1816780)
Supplement: Supplementary file 2 [file Table_2.docx]

Table S2. Dominant fungal species detected per high and low probability infection, per hip or knee

| **Fungi** | **Overall** N = 26 | **High_Hip** N = 0 | **High_Knee** N = 10 | **Low_Hip** N = 1 | **Low_Knee** N = 15 |
| --- | --- | --- | --- | --- | --- |
| *Candida parapsilosis* | 6 (23%) | 0 (0%) | 1 (10%) | 0 (0%) | 5 (33%) |
| *Candida albicans* | 5 (19%) | 0 (0%) | 3 (30%) | 1 (100%) | 1 (6.7%) |
| *Aureobasidium pullulans* | 3 (12%) | 0 (0%) | 2 (20%) | 0 (0%) | 1 (6.7%) |
| *Candida glabrata* | 3 (12%) | 0 (0%) | 2 (20%) | 0 (0%) | 1 (6.7%) |
| *Aspergillus vitricola* | 1 (3.8%) | 0 (0%) | 0 (0%) | 0 (0%) | 1 (6.7%) |
| *Candida metapsilosis* | 1 (3.8%) | 0 (0%) | 0 (0%) | 0 (0%) | 1 (6.7%) |
| *Candida orthopsilosis* | 1 (3.8%) | 0 (0%) | 0 (0%) | 0 (0%) | 1 (6.7%) |
| *Cladosporium halotolerans* | 1 (3.8%) | 0 (0%) | 0 (0%) | 0 (0%) | 1 (6.7%) |
| *Coccidioides posadasii* | 1 (3.8%) | 0 (0%) | 0 (0%) | 0 (0%) | 1 (6.7%) |
| *Malassezia globosa* | 1 (3.8%) | 0 (0%) | 1 (10%) | 0 (0%) | 0 (0%) |
| *Malassezia restrica* | 1 (3.8%) | 0 (0%) | 0 (0%) | 0 (0%) | 1 (6.7%) |
| *Naganishia diffluens* | 1 (3.8%) | 0 (0%) | 1 (10%) | 0 (0%) | 0 (0%) |
| *Penicillium sp* | 1 (3.8%) | 0 (0%) | 0 (0%) | 0 (0%) | 1 (6.7%) |
|  |  |  |  |  |  |
